# Quantitative Gait Analysis Detects Significant Differences in Movement between Osteoarthritic and Nonosteoarthritic Guinea Pig Strains before and after Treatment with Flunixin Meglumine

**DOI:** 10.1155/2014/503519

**Published:** 2014-05-19

**Authors:** K. S. Santangelo, A. C. Kaeding, S. A. Baker, A. L. Bertone

**Affiliations:** ^1^Department of Veterinary Biosciences, The Ohio State University, Columbus, OH 43210, USA; ^2^Department of Microbiology, Immunology, and Pathology, College of Veterinary Medicine & Biomedical Sciences, Colorado State University, Fort Collins, CO 80523, USA; ^3^Department of Veterinary Preventative Medicine, The Ohio State University, Columbus, OH 43210, USA; ^4^Department of Veterinary Clinical Sciences, The Ohio State University, Columbus, OH 43210, USA

## Abstract

A computer-aided gait analysis system was used to contrast two guinea pig strains with differing propensity for osteoarthritis (OA), with/without administration of a nonsteroidal anti-inflammatory drug. Walking speed and static/dynamic gait parameters were determined at baseline. Flunixin meglumine was given and animals were evaluated 4, 24, and 72 hours after treatment. Body weight was compared using unpaired *t*-tests. Knee joints were histologically evaluated using species-specific criteria; indices were analyzed using one-way ANOVA, Kruskal-Wallis test, followed by Dunn's multiple comparisons. A generalized linear model followed by Tukey's posttests juxtaposed gait parameters; walking speed was a covariate for other outcome measures. Body weight was not different between strains; OA-prone animals demonstrated more progressive chondropathy. At baseline, OA-prone animals had slower walking speeds, narrower hind limb bases of support, shorter stride lengths, and slower limb swing speeds relative to OA-resistant animals. These differences were not detected 4 or 24 hours after treatment. By 72 hours, OA-prone animals had returned to baseline values. These findings indicate a distinct voluntary gait pattern in a rodent model of bilateral primary OA, modification of which may allow rapid screening of novel therapies. Flunixin meglumine temporarily permitted OA-prone animals to move in a manner that was analogous to OA-resistant animals.

## 1. Introduction

The major clinical symptom associated with osteoarthritis (OA) is pain during motion. Unfortunately, the severity of joint disease determined via imaging and histology is often only weakly associated with the chief complaint [1]. As disturbances in movement are known consequences of pain [2], analyses of gait in parallel with standard measures of pathology have started to find favor with animal models of human disease. Techniques utilized to evaluate OA in rodents include assessment of musculoskeletal function (grip strength meters, rotarod testing) and gait and spontaneous activity testing (force platforms, video-recorded open-field and arena trials) [3]. Walkway- [4] and treadmill-associated [5] computer-aided gait analysis systems have also been validated to gauge gait abnormalities in injury-induced models of arthritis.

An automated quantitative gait analysis method, the Noldus CatWalk, determines a large number of locomotion parameters in laboratory animals during spontaneous, unforced platform crossing [6]. This computer-assisted method objectively and rapidly monitors a large number of both dynamic and static gait statistics, including duration of different phases of the step cycle and pressure applied during locomotion. Recently, this system has documented gait abnormalities associated with rheumatoid arthritis [7] and OA [8] and has been shown to be reliable for clinically relevant evaluation of movement-related nociception, the hallmark of OA-induced pain [9]. While this system has been used to study secondary OA in rodent models, it has not been employed with a primary model of OA.

As there is no artificial derangement of the joint, the Hartley guinea pig model of naturally occurring OA is an attractive option for studying disease onset and progression. Starting at 3 months of age, this strain of guinea pig develops OA of the knee that bears histological and biochemical resemblance to pathology seen in humans, with lesions appearing predominantly on the medial side of the joint [10]. In regard to gait, this OA-prone strain exhibits peripheral sensitization such that joint movements, even in the normal working range, elicit burst activity of primary afferent neurons with resultant pain [11]. Of equal relevance, an OA-resistant strain, Strain 13, has been employed for comparison purposes. This strain—although not completely free of OA—develops significantly less severe histology disease than the Hartley strain, with only mild joint changes present at 12 months of age [12]. Work using the CatWalk with any strain of guinea pig has not been published.

Nonsteroidal anti-inflammatory drugs (NSAIDS) are frequently administered arthritis medications due to their analgesic and anti-inflammatory properties [13]. Currently, there are relatively few papers that evaluate NSAID administration on gait parameters in rodents; those that do involve rheumatoid arthritis [4, 14, 15] and injury-induced OA [8, 9]. Although not prescribed for humans, flunixin meglumine—a nonselective cyclooxygenase (COX) inhibitor—has demonstrated efficacy for treating musculoskeletal conditions in the veterinary market, particularly in equine sports medicine. In horses and other species, onset of action following single administration occurs within 2 hours and can persist up to 30 hours [16]. While this NSAID is a common preemptive and postoperative analgesic agent utilized in guinea pigs, the effectiveness of flunixin meglumine has not been evaluated in this, or other, rodent models of joint degeneration.

Our study addressed two hypotheses: (1) significant differences in baseline gait parameters exist between guinea pig strains with varying propensity for OA and (2) gait parameters of OA-prone Hartley guinea pigs will be statistically altered following a single standard dose of flunixin meglumine. Aged Hartley guinea pigs with advanced OA were utilized to provide the highest amount of clinical relevance and the greatest challenge to the selected NSAID. In addition to baseline measurements, time points of 4, 24, and 72 hours after NSAID administration were chosen based upon the drug's typical onset and duration of action, as described above.

## 2. Materials and Methods 

Procedures were approved by the university's Institutional Laboratory Animal Care and Use Committee and were performed in accordance with the NIH Guide for the Care and Use of Laboratory Animals. Four male OA-prone Hartley guinea pigs were obtained from Charles River Laboratories (Wilmington, MA, USA) and 4 OA-resistant male Strain 13 animals from the US Army Medical Research Institute of Infectious Diseases (Fort Detrick, MD, USA) for data collection at 480 days of age. Animals were housed in groups of two in solid bottom cages and allowed* ad libitum* water and guinea pig chow (Harlan Teklad 7006) containing vitamin C (800 mg/kg) and vitamin D3 (2.4 IU/g). Body weight (grams) was monitored throughout the study and is reported as mean ± 95% confidence intervals.

Animals were acclimated to the gait analysis system (Noldus Catwalk 7.1; Leesburg, VA, USA) over 1 week. Incentive for walking across the platform included the presence of a small amount of food (a mixture of lettuce, apple, and carrot) and the animal's cage mate. Training and data collection were performed during the same period (10 AM to 2 PM) and involved the same handlers. All procedures were executed in the dark (except for light emitting from the nearby computer screen), the environment in which animals were most willing to walk on the elevated platform. The order in which animals were selected to undergo data collection for each time point was assigned in a random fashion.

The gait analysis system has been described [6–9]. Briefly, subjects traversed a glass plate walkway (100 cm × 15 cm × 0.6 cm) through a 17.6 cm wide tunnel. LED light entered the distal long edge of this glass floor from an encased fluorescent lamp and was internally reflected, scattering at points where paws touched the glass. A high-speed color video camera (camera frame rate = 100 fps; CCD imager = 0.5′VGA; S/N ratio > 58 dB) was located 44 cm beneath the apparatus and recorded the digitized signal for computer-based examination (CatWalk Software 7.1). For data analysis, software automatically labeled all illuminated areas containing pixels above the set threshold of 7 pixels/arbitrary units. The threshold was then set at 40 arbitrary units (within a possible range of 0 to 255) to minimize any potential background [2, 4, 8]. The area of a single pixel was 1 mm^2^/pixel^2^. Regions correlated to paw prints were identified and assigned to respective limbs. The average values from three complete, uninterrupted runs (walking speed ≥ 15 cm/s; ≥4 complete step cycles per limb) were analyzed for each animal at designated time points; as such, a total of 96 runs are represented in this study. Emphasized gait parameters included* walking speed* (cm/sec), distance crossed by the animal in the corridor divided by the time taken to complete the distance;* base of support* (cm), average width between either the front or hind paws (as measured from the mean *Y* value of each paw, with the major body axis of the animal, on average, aligned in the *X* direction);* stride length* (cm), distance between successive placements of the same paw (as measured from the mass midpoint of each paw); and* swing speed* (cm/s), stride length divided by the swing phase duration. Gait analysis data are represented as mean ± 95% confidence intervals. Hind limb comparisons are provided for* base of support*. One-way ANOVA did not reveal significant statistical differences among limbs for* stride length* and* swing speed*; combined data for all four limbs was supplied.

Following a 7-day acclimation to the apparatus, baseline paw statistics were collected for all guinea pigs. Two days later, a standard analgesic dose of 5 mg/kg flunixin meglumine (Merck Animal Health, Whitehouse Station, NJ, USA) at a concentration of 5 mg/mL (diluted from the commercially available 50 mg/mL stock with sterile water for injection, USP) was administered once subcutaneously between the shoulder blades. Four, 24, and 72 hours after administration, animals were run on the gait system for additional data collection and comparison.

Animals were euthanized following study completion via CO_2_ inhalation followed by bilateral thoracotomy. Both hind limb knees from each animal were evaluated using the OA scoring system described below [10, 17]. Whole knee joints were fixed in 10% neutral buffered formalin and prepared for histological analysis, as previously described [17]. Paraffin sections (5 *μ*m) were taken from the center of the medial tibial plateau in each joint and stained with toluidine blue. Two independent, blinded observers (KSS, ALB) performed histological grading of serial coronal sections of each knee, using adapted Mankin criteria based upon species-specific features. Histological evidence of chondropathy incorporated (1) grading of articular cartilage structure from 0 to 8 and (2) grading of proteoglycan loss, as determined by loss of toluidine blue staining intensity, from 0 to 6. Chondropathy was scored for medial and lateral tibial plateaus. As Wilcoxon matched pairs tests did not reveal significant differences between left and right knees for either guinea pig strain, a finding corroborated by other studies, indices were averaged between both limbs [12, 17]. Total tibial indices ranged from 0 (normal) to 28 (severe structural damage and complete loss of toluidine blue staining) and are shown as median (minimum, maximum).

Body weights were analyzed using an unpaired *t*-test. OA indices were analyzed using one-way ANOVA, Kruskal-Wallis test, followed by Dunn's multiple comparison post hoc test [17]. These analyses were performed using GraphPad Prism Version 4.0 (San Diego, CA, USA) with a statistical significance of *P* < 0.05. Statistics for gait parameters were completed using SAS Version 9.3 (Cary, NC, USA); *P* < 0.05 was considered statistically significant. Walking speed was analyzed using a generalized linear model with two categorical factors (strain and time) followed by Tukey's posttests. As base of support, stride length, and swing speed are correlated with an animal's selected velocity, walking speed was included as a covariate in these analyses [18]. For each individual gait parameter, a generalized linear model (as above, followed by Tukey's posttests) with a linear dependence on walking speed was utilized to assess statistical differences. When applicable, estimated differences (ED) in means and *P* values are provided.

## 3. Results

**Figure 1:**
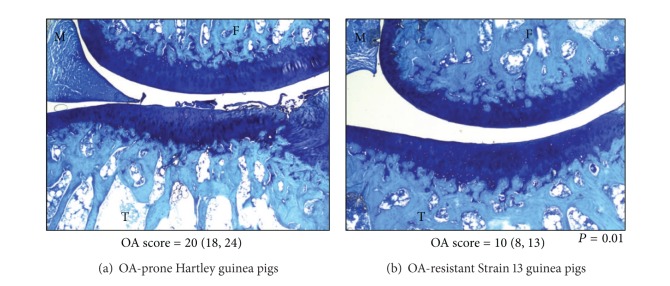
Representative photomicrographs demonstrating toluidine blue stained knee joints (100×, final magnification) taken from OA-prone Hartley guinea pigs (a) and OA-resistant Strain 13 guinea pigs (b). The medial knee compartment is shown: M: menisci; F: femur; and T: tibia. OA scores are reported as median (minimum, maximum). Data was analyzed using one-way ANOVA, Kruskal-Wallis test, followed by Dunn's multiple comparison post hoc test. As expected, a significant statistical difference (*P* = 0.01) in chondropathy is present between the two guinea pig strains.

A significant statistical difference in body weight (grams) was not detected between OA-prone (971.8 ± 103.3) and OA-resistant (1035.0 ± 104.2) animals, a finding that is consistent with published work [12, 17]. As previously described [12, 17], a significant statistical difference (*P* = 0.01) in chondropathy was present between OA-prone (20 (18, 24)) and OA-resistant (10 (8, 13)) animals (Figure 1).

**Figure 2:**
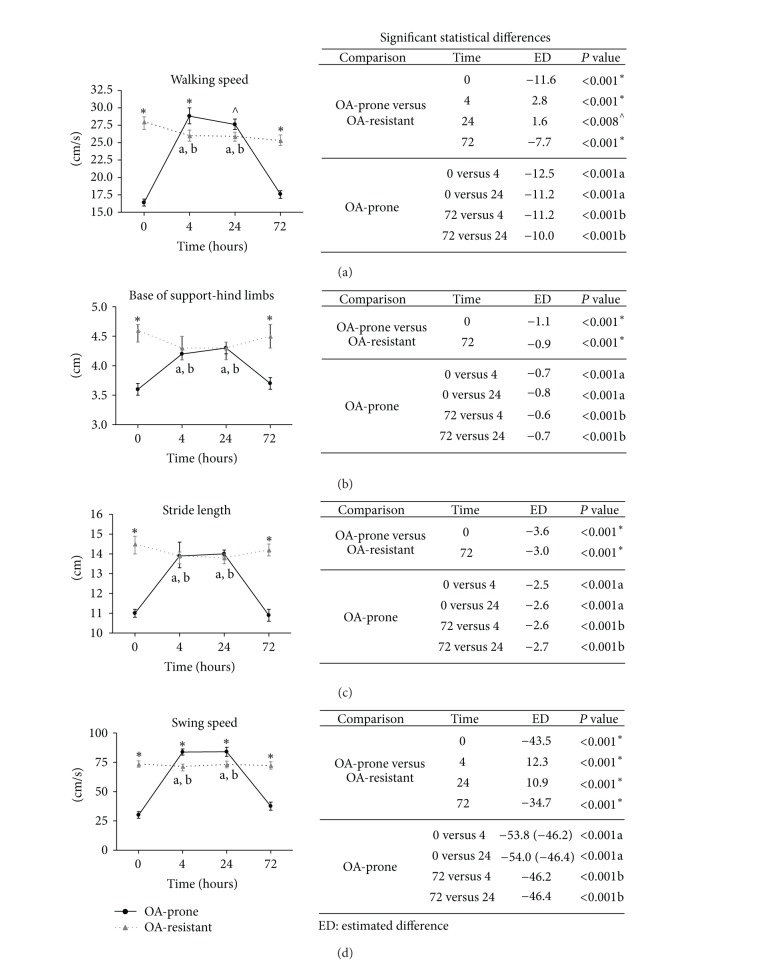
Mean values ± 95% confidence intervals for indicated gait analysis data for two guinea pig strains with varying propensity for osteoarthritis (OA-prone and OA-resistant). Data were evaluated in both strains prior to administration (baseline, time 0) of flunixin meglumine, a nonsteroidal anti-inflammatory drug (NSAID), as well as at 4, 24, and 72 hours after administration. Significant statistical differences were determined via a generalized linear model followed by Tukey's posttests; walking speed was included as a covariate in the analyses performed on other gait parameters. *P* values <0.05 are provided, as well as estimated differences (ED) of the means.

**Figure 3:**
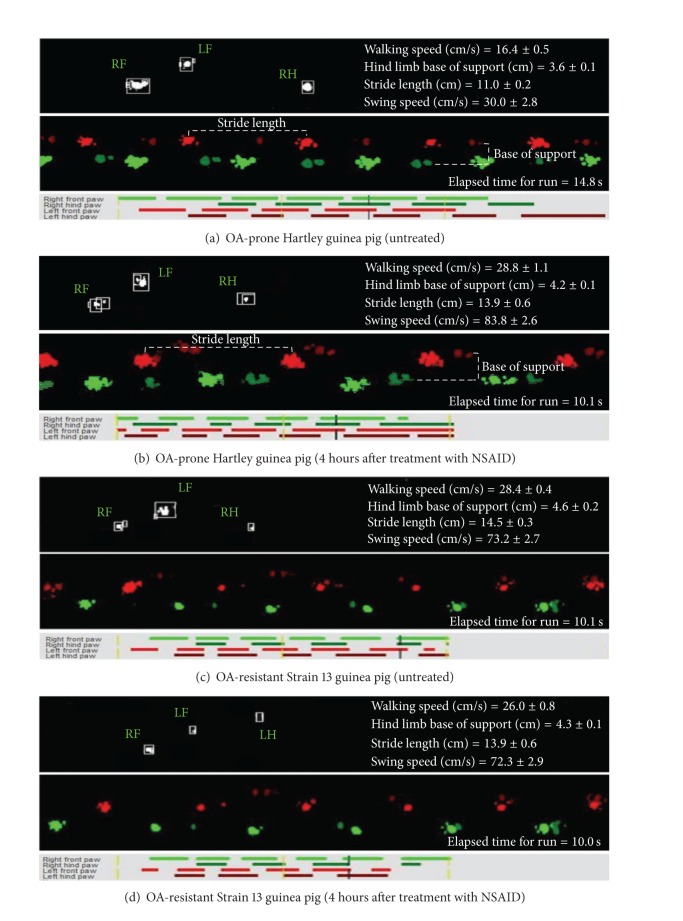
Representative still images of data output provided by the computer-aided gait analysis system for one OA-prone Hartley guinea pig and one OA-resistant Strain 13 guinea pig at baseline and 4 hours after treatment. Top images of each data set are photographs of streaming video footage collected by the underlying camera. Computer-constructed paw prints utilized for data analysis are presented in the middle image. Bottom images show the length of time when each individual paw is in contact with the glass surface. Time (in 5-second intervals) is denoted by vertical yellow, dashed lines; vertical black, dashed lines designate where live videos correspond to individual paw prints. Paw prints are color-coded, as indicated. As walking is a four-beat gait, three paw prints are in contact with the ground at any one time. Elapsed time of each particular run and gait parameters (mean ± 95% confidence intervals) for each group are provided. At 4 (shown) and 24 hours after treatment, single administration of flunixin meglumine permitted OA-prone animals to move with a gait profile that was not significantly different from OA-resistant animals.

Gait parameters from OA-prone guinea pigs were statistically different from OA-resistant animals and were also significantly different after administration of flunixin meglumine (Figures 2 and 3). Specific outcome measures of interest are addressed below.

Walking speed (Figure 2(a)): OA-prone guinea pigs at baseline and 72 hours had statistically (*P* < 0.001) slower walking speeds than OA-resistant animals (ED = −11.6 and −7.7 cm/s, resp.). The walking speeds of OA-prone guinea pigs at 4 and 24 hours after NSAID, however, were significantly (*P* ≤ 0.008) increased compared to baseline (ED = 12.5 and 11.2 cm/s, resp.) and relative to OA-resistant animals (ED = 2.8 and 1.6 cm/s, resp.). The walking speeds of OA-resistant animals at 4, 24, and 72 hours were statistically (*P* ≤ 0.001) slower than at baseline (ED = 2.4, 2.4, and 3.1 cm/s, resp.).

Base of support (Figure 2(b)): at baseline, OA-prone guinea pigs demonstrated significantly (*P* < 0.001) narrower hind limb bases of the support relative to OA-resistant animals (ED = −1.1 cm). Four and 24 hours after drug administration, hind limb base of support in OA-prone guinea pigs was statistically (*P* < 0.001) wider than baseline (ED = 0.7 and 0.8, resp.) and was not statistically different from OA-resistant animals. Similar to baseline values, OA-prone animals 72 hours after treatment had significantly (*P* < 0.001) narrower hind limb bases of support relative to OA-resistant animals (ED = −0.9 cm).

Stride length (Figure 2(c)): OA-prone guinea pigs had statistically (*P* < 0.001) shorter stride lengths than OA-resistant animals at baseline (ED = −3.6 cm). Four and 24 hours following NSAID, OA-prone guinea pigs were significantly (*P* < 0.001) longer than baseline values (ED = 2.5 and 2.6 cm, resp.) and did not show statistical differences from OA-resistant animals. Seventy-two hours later, stride lengths of OA-prone animals were not significantly different from baseline.

Swing speed (Figure 2(d)): at baseline, OA-prone guinea pigs had significantly (*P* < 0.001) slower swing speeds than OA-resistant animals (ED = −43.5 cm/s). Four and 24 hours following NSAID, OA-prone guinea pigs had statistically (*P* < 0.001) faster swing speeds than baseline values (ED = 53.8 and 54 cm/s, resp.) and were significantly (*P* < 0.001) increased relative to OA-resistant animals (ED = 12.3 and 10.9, resp.). Seventy-two hours later, swing speeds of OA-prone guinea pigs were not statistically different from baseline values.

## 4. Discussion

This proof-of-principle report is the first to document use of the CatWalk system in a spontaneous model of OA, as well as its application with guinea pigs. With minor adjustment of camera placement (44 cm below the walking platform for guinea pigs versus 42 cm for rats), this technique easily accommodated a larger rodent species with no adverse influence on the quality of data collection. Computer-aided gait analysis was able to provide a wide array of outcome measures that would be challenging to reveal without video and computer assistance. Further, use of this technique distinguished significant statistical differences in pertinent gait parameters with a relatively low number of animals.

This computer-aided system has been useful in identifying alterations in gait parameters in unilateral, injury-induced models of OA. Differences in static paw statistics, including paw pressure (light intensity) and print area [9, 19–21], and dynamic gait parameters, such as swing speed, stance phase, duty cycle, and interlimb coordination [2, 4, 8, 22], have been documented and logically reflect the one-sided nature of the arthritis induced in the experiments. Changes in paw pressure, print area, and interlimb coordination were neither observed in our work—in regard to either strain or treatment—nor were they anticipated given the bilateral nature of disease in this guinea pig model.

Our study demonstrated that aged OA-prone guinea pigs had narrower hind limb bases of support, shorter stride lengths, and slower swing speeds, which are likely attributed to the overall slower walking speed of this strain relative to OA-resistant animals. To account for this, walking speed was included as a covariate in the statistical analyses for these gait parameters [18]. In humans, increased knee joint nociception results in gait adaptations to minimize pain sensation by lowering biomechanical articular stress in affected limbs [23]. Given that OA was present in both knees of Hartley guinea pigs, we hypothesize that these animals moved slowly to avoid discomfort and/or compensate for decreased physical/mechanical mobility. When this hindrance was temporarily alleviated by use of flunixin meglumine, these animals voluntarily moved at speed and, hence, in a manner similar to that of OA-resistant animals.

As expected, treatment with an NSAID did not alter the majority of gait parameters measured in OA-resistant Strain 13 animals. A statistically significant decrease in walking speed of OA-resistant guinea pigs was present 4, 24, and 72 hours after administration of flunixin meglumine, but this trend is unlikely to be attributed to therapeutic effect. Rather, we propose that the motivation provided to these animals during the instrument acclimation period, including food and companionship with their cage mate, became less appealing over time. Of note, this adds further credence to the influence flunixin meglumine had on OA-prone animals, which did not demonstrate this same decline in impetus. Indeed, the most striking subjective findings in our study were the increased ease and willingness of the treated OA-prone animals to walk—and walk quickly—even when typical sources of reward were removed.

As single administration of flunixin meglumine demonstrated efficacy in improving the gait of OA-prone Hartley guinea pigs, further effort is warranted to determine the central and/or peripheral mechanism(s) of action that contributed to enhanced movement. In a project that evaluated the rat model of unilateral OA induced by articular injection of monoiodoacetate (MIA) via this same gait analysis system, treatment with celecoxib (a selective COX-2 inhibitor) did not result in a significant statistical difference in walking speed but did cause early improvement in swing speed and duty cycle [8]. The authors suggested that celecoxib appears to work in the early inflammatory phase of injury that is associated with central sensitization, but not in the local, chronic pain phase of the affected articulation. Another CatWalk-based study assessed diclofenac (also a COX-2 inhibitor) in this same MIA model and found a marked antinociceptive effect only during the initial inflammatory state (day 3) [9]. Given the challenge in directly drawing correlations regarding our own work from these related yet different models, projects focused on determining the potential benefits of flunixin meglumine and other selective and nonselective NSAIDs on nociception in this primary model of OA are planned. In particular, it would be interesting to compare profiles of inflammatory and pain mediators, both circulating and local to the joint environment, before and after NSAID administration.

We acknowledge that utilizing a higher number of animals may have revealed important changes in gait parameters not shown in the current paper. However, the relevance of successfully detecting the highly significant differences that were present between and among groups over time with a lower sample size should not be underappreciated. Indeed, this work was intended to provide stringent challenges for both the gait analysis system and NSAID to overcome, and both proved to be effective. As such, these results demonstrate that* in vivo* screening of novel therapeutic agents can be achieved using these methods with a small number of subjects over multiple time points without animal sacrifice, which are applicable findings for both academia and industry.

## 5. Conclusion

In summary, utilization of a quantitative analysis system elucidated gait parameters that are indicative of bilateral knee OA and likely reflective of a slower voluntary walking speed. Significantly, treating Hartley guinea pigs with flunixin meglumine permitted these OA-prone animals to move with a gait pattern that was comparable to OA-resistant Strain 13 animals. As such, this study may provide a foundation for rapid* in vivo* screening of novel treatment modalities on a relatively small number of animals with naturally occurring OA.
